# Migration of disrupted sternal wire to the pulmonary artery

**DOI:** 10.1186/s44215-025-00207-4

**Published:** 2025-04-07

**Authors:** Yuji Naito, Fumitaka Suzuki, Tatsuya Murakami

**Affiliations:** Department of Thoracic Surgery, Asahikawa City Hospital, 1-65, Kinsei-Cho 1, Asahikawa, Hokkaido 070-8610 Japan

**Keywords:** Sternal wire, Foreign body, Migration, Disruption

## Abstract

We report a case of sternal wire migration into the pulmonary artery. A 66-year-old man who had undergone thymectomy through median sternotomy 3 years ago was admitted because of a fractured sternal wire in the right pulmonary artery on computed tomography during the postoperative follow-up. It was removed directly from the pulmonary artery under cardiopulmonary bypass. The postoperative course was uneventful. Although migrated sternal wire into the heart or vascular tissue is very rare, care is necessary for its disruption and displacement after sternotomy.

## Background

Disruption of the sternal wire used for sternal closure after sternotomy is occasionally observed in patients with sternal instability, but migration of disrupted wire into the heart or vascular system occurs rarely. We report a case in which the sternal wire used in previous sternotomy was disrupted, migrated to the pulmonary artery, and successfully removed.

## Case presentation

**Fig. 1:**
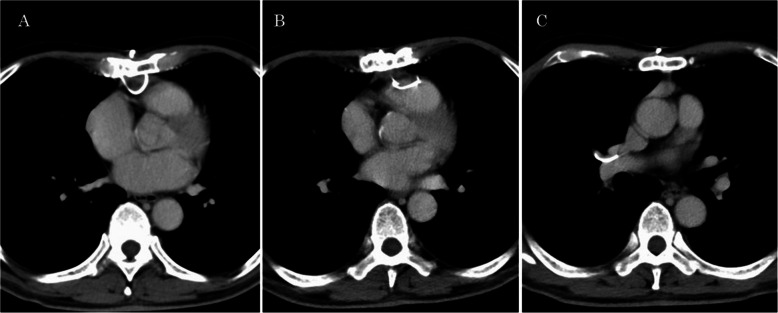
CT images performed after the initial operation. **A** Image after 6 months after the surgery. Redundant part of the sternal wire not securely fastened protrudes into the heart. **B** Image after 2 years. A fractured wire is observed in or on the right ventricle. **C** Image after 3 years. A fragment of the wire is in the right pulmonary artery

**Fig. 2:**
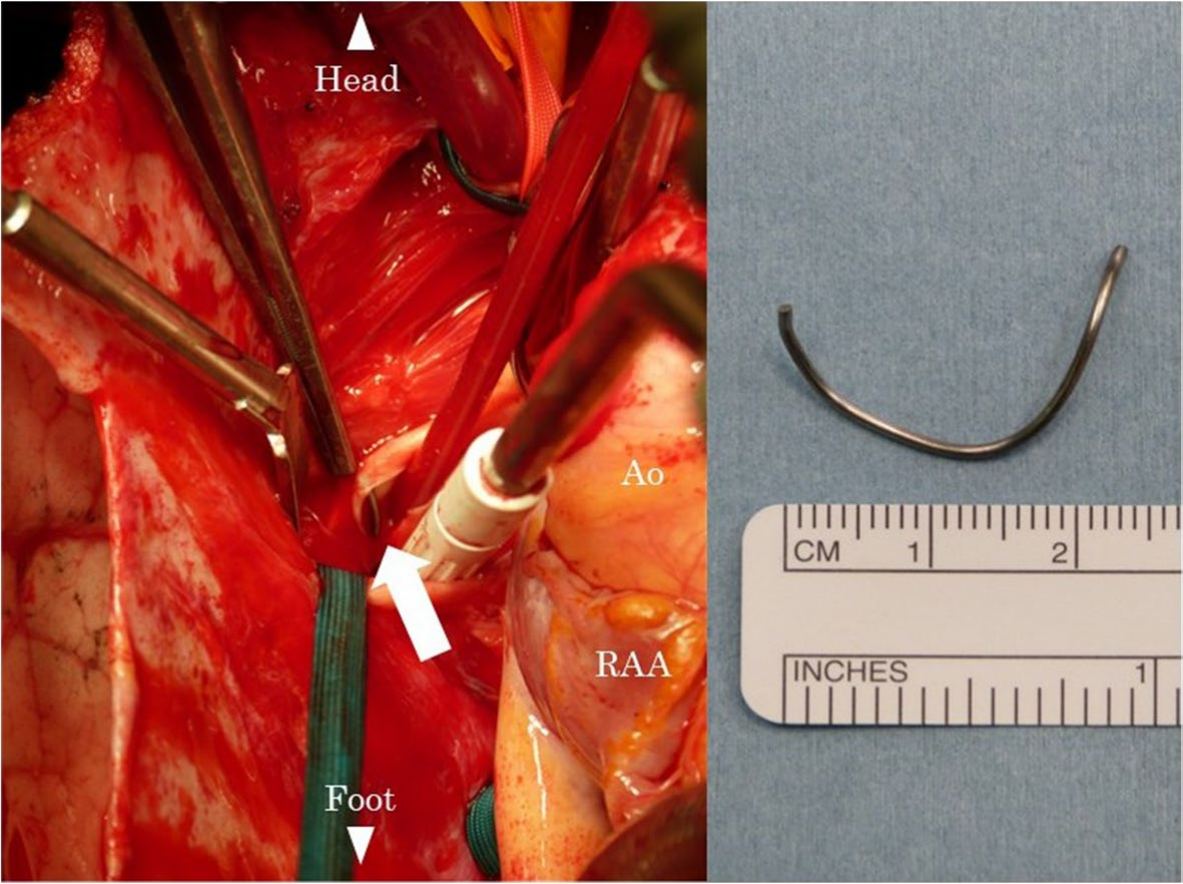
Intraoperative images after pulmonary arteriotomy (left). The arrow indicates a fragment of the sternal wire in the right lower pulmonary artery. Sternal wire fragment removed from the pulmonary artery (right). Ao, aorta; RA, right atrial appendage

A 66-year-old man without symptoms who had undergone thymectomy through sternotomy 3 years previously was introduced for us because of migration of the fractured sternal wire into the right pulmonary artery. Computed tomography (CT) showed that 4th sternal wire of all 5 wires disrupted and the fractured wire was located in the right middle and lower pulmonary artery (Fig. 1). There was no sternal instability. On retrograde inspection, CT evaluation 6 months after the initial thymectomy surgery revealed that the wire was not completely fastened, and the redundant part behind the sternum protruded into the heart. CT 2 years later showed that the redundant part was disrupted and the fracture of it was in front of, or in the muscle of the right ventricle (Fig. 1). It was estimated that the redundant part of the wire was disrupted by heart pulsation for a long time and then migrated through the right ventricle muscle and into the cavity and flowed to the right pulmonary artery. There was a risk of pulmonary thromboembolism and hemorrhage if the fracture wire was left there. Then, surgical removal was performed. After re-sternotomy, there was light adhesion between the pericardium and right ventricle, which might have been where the fragment passed through the right ventricular wall. Cardiopulmonary bypass was established using aortic and superior and inferior vena cava cannulation. Dissection of the right hilum revealed the middle and lower branches of the right pulmonary artery. The wire was seen transparently in the artery. The wire was removed via a small arteriotomy without cardiac arrest (Fig. 2). The postoperative course was uneventful, and the patient was discharged 14 days after surgery.

## Discussion

The many foreign bodies in the pulmonary artery are catheter fragments. The removal procedure is performed due to the risk of thromboembolism, infection, and hemorrhage. Typically, removal is performed via an endovascular procedure. It is good if the foreign body is soft material, but it is dangerous that a hard and steep material, like a sternal wire fragment in this case, is dragged in the heart and/or vessel because the material can damage the structure and cause hemorrhage. Then, we performed open surgery under cardiopulmonary bypass.

This case required open-heart surgery because a fragment of the wire was lodged in the pulmonary artery. However, removal without cardiopulmonary bypass might have been possible 2 years after the initial operation, when the fragment was in the right ventricle, if it had been located on the epicardium rather than in the right ventricle wall or cavity. On the other hand, it would have been difficult to consider a removal procedure when the wire was not fragmented, but was only protruding into the heart, 6 months after the surgery. It was unclear from the CT image at that time whether it was embedded in the ventricular wall or merely pressing against it. Echocardiography could have been useful for that evaluation. Peripheral cardiopulmonary bypass would have been required before re-sternotomy and removal of the wire if there had been a possibility that it was embedded in the heart.

Disruption of the sternal wire after sternotomy occurs occasionally in the presence of sternal instability. Boiselle et al. [1] reported that 21% of patients with sternal dehiscence experienced wire disruption. However, it is infrequent that a sternal wire is disrupted and fragmented. Schreffler et al. [2] reported the only case in which the sternal wire migrated to the pulmonary artery and caused pulmonary hemorrhage by penetrating the bronchus. Others reported a case of cardiac tamponade caused by a wire fragment injuring the heart [3], and a case was reported in which a wire migrated into the muscle of the right ventricle without any bleeding event and was removed [4]. In this case, the disruption of the wire occurred because the redundant part behind the sternum due to incomplete fastening during primary surgery was shaken for a long time by pulsation of the heart.

## Conclusion

We report a case of removal of a sternal wire fragment that had migrated to the pulmonary artery. Care should be taken with the sternal wire during follow-up after sternotomy.

## Data Availability

The datasets used during the current study are available from the corresponding author on reasonable request.
